# Buffalo Pathological Society

**Published:** 1890-02

**Authors:** 


### Society Proceedings.

### BUFFALO PATHOLOGICAL SOCIETY.

### REGULAR MEETING, DEC. 20, 1889.

THE President, Dr. DE LANCEY ROCHESTER, in the chair.

Before the regular routine of business, Dr. HERMAN MYNTER presented a patient upon whom he had made a partial resection of the foot for pes varo-equinus, with an excellent result.

The patient, Thomas Chaffee, aged 23, entered the Sisters' Hospital on Oct. 8, 1889, in order to have his leg amputated on account of a pes varo-equinus of the highest degree, complicated with an ulcerated bunion, which had prevented him from walking for the last five years. When five years of age he was run over by a sleigh, producing a dislocation of left ankle. A physician, he says, cut the outer cord. Contraction of the tibialis anticus muscle and of the tendo achilles followed, increasing during the year till he now walks on the dorsum of the foot, which is twisted in such a degree that the big toe almost touches the internal malleolus. During the last five years he has been unable to walk on account of a suppurating bunion of the dorsum of the foot, on the most declining point. If he walks for one week he will have to stay in bed for several months to have the ulcer heal again. He has consulted seven physicians, who all advised amputation.

On Oct. 12, 1889, operation under ether narcosis. An incision was made along the anterior margin of the foot from the anterior part of the calcaneus to the middle of the fifth metatarsal bone. From the posterior end of this a transverse incision was made over the dorsum of the foot to the scaphoid. The extensor tendons were lifted away with blunt instruments, and with chisel and Wyeth's osteotome a wedge of bone was removed from the anterior side of the foot, containing the anterior three-fourths of the cuboid, second and third cuneiform bones, and the bases of fifth, fourth, and third metatarsal bones. Having in that way shortened the anterior margin, the inner margin of the foot was lengthened by a Phelps operation in front of the malleolus internus, by which the following structures were cut over: Tibialis anticus, tibialis posticus, flexors halluces longus and brevis, abductor hallucis and fascia plantaris. The joints between the astragalus and scaphoid was thereafter widely opened. Lastly, tenotomy of the tendo Achiles was performed. The foot was now, by aid of considerable force, brought in a good position, the wounds sutured with catgut except in the middle, left open for drainage, a strict antiseptic bandage applied, and over that a plaster of Paris dressing. Lastly, the Esmarch was removed and the wounds filled with an antiseptic Schede's blood-clot. The foot was kept raised for 24 hours. Very slight surgical fever followed and scarcely any pain. In six weeks the first dressing was removed and the anterior wound found healed. At the point of the Phelps operation a granulating blood-clot was seen, not yet covered with epithelium. In three weeks more the wound was healed, and with a Kolbe's shoe the patient was then able to walk without pain.

Discharged, cured, with the foot in excellent condition, on December 19th.

Dr. Wm. C. Krauss read the first of three papers on Peritonitis, as follows:

### THE PATHOGENESIS AND MORBID ANATOMY OF PERITONITIS.

By WM. C. KRAUSS, M. D.

The subject open for discussion this evening, is that of Peritonitis,—a condition teeming with expanse and limits of observation, accuracies and errors of diagnosis, facilities and perplexities of treatment—all dependent upon the observer.

Few, if any, of the inflammatory changes can be approached from so many sides, as the inflammatory state of the peritoneum. The physician, surgeon, gynecologist, and obstetrician, find in it a field rich in thought and action. Through its agency many have signaled success and renown, and among this number America is credited with some of the first and foremost.

Peritonitis is an inflammatory condition of the serous membrane of the abdominal cavity. It may be general or localized; when localized, it takes the name of the viscus in which resides the inflammation. An acute local peritonitis may become rapidly general, or it may remain circumscribed and terminate in resolution. Peritonitis may occur at any age, and at any time, spares neither the robust nor feeble, knows no sex, and no station.

The etiology of peritonitis varies according to the insult. That insult may be purely medical, as in perityphlitis with ulceration; surgical, or traumatic, as in gunshot wounds of the abdomen ; gynecic, as in the rupture of ovarian cysts ; parturient, as seen in septic processes, as puerperal fever.

Peritonitis, due to a medical insult, may be acute or chronic. The acute form may be either idiopathic or secondary. Idiopathic peritonitis is, however, of very rare occurrence. In those cases where a definite cause is unrecognizable, it is termed idiopathic, yet, if an autopsy were made, fully ninety-five per cent. of such cases could be classified under secondary peritonitis. The etiology of such cases, if such occur, has been ascribed to sudden changes in the temperature of the intestine, excesses in food and drink, intestinal irritants, etc.

The secondary form claims most of our attention. Here the causes are legion. A careful study of the tissues and neighboring tissues, organs and neighboring organs, of the abdominal cavity,—in fact the whole of human anatomy, with the possible exception of the cranium and extremities,—is necessary for a perfect comprehension of what *might* and *has* produced an inflammatory condition of the peritoneum.

Among the most prolific causes of peritonitis due to a medical insult, are lesions of the intestinal canal. As such, perhaps, none are so productive as perforating ulcers of typhoid fever, and ulcerating typhlitis and perityphlitis. Other causes may be mentioned, such as proctitis and periproctitis ; syphilitic, or tubercular ulcers of the intestine ; strangulated hernias, intestinal intussusception, volvuli, impaction, etc. These are some of the affections of the intestinal canal, which, through perforation, ulceration, or extension of the inflammation, may encroach upon the peritoneum. Among the most fruitful of hepatic disorders, are hepatitis and perihepatitis, rupture of hydatid cysts, obstruction and rupture of the gall-bladder or duct, cirrhotic conditions, and neoplasms. Inflammatory conditions of other organs as splenitis, pancreatitis, nephritis, cystitis, cancerous, syphilitic or tubercular neoplasms of the various organs, ruptures, as of the uterus, bladder, abdominal, aneurisms, receptaculum chyli, hydatid and other cysts. These all may set up peritoneal inflammations. In children, gangrenous inflammation of the umbilical vessels, and incomplete descent of the testicles have been known to produce inflammatory states. During the course of septic and other processes,—as septicemia, pyemia, erysipelas, rheumatism, etc.,—the peritoneum is occasionally drawn into sympathy.

Surgical or traumatic insults play an important role, and are among the most fertile factors in effecting a peritonitis. Here judgment, above all, is requisite on the part of the observer, in recognizing the probability and consequence of trauma as an etiological factor.

Gunshot and incised wounds of the abdomen are of too frequent occurrence to need comment. Direct trauma to the abdomen resulting in the rupture of some internal organ, unsuccessful or blundering laparatomies, the rupture of abscesses due to caries of the vertebræ, ribs or pelvis, are some of the surgical agencies which must not be omitted in this connection.

Gynecic insults are more or less local, calling forth a pelvic peritonitis, which very often becomes general. As such, gonorrheal infection may be considered as most habitual and most universal. Rupture of ovarian cysts, ovarian tumors, the extension of uterine inflammations, injection of irritant fluids through the Fallopian tubes, uterine congestion following disordered menstruation, lymphangitis and phlebitis of the uterus are some of the recognized causes of pelvic peritonitis.

Parturient insults are among the most obstinate, most common, and saddest sources of this inflammatory process. Here the enactment of physiological laws terminates in pathological conditions and sequels. Foremost among the parturient causes is the septic process designated puerperal fever, rising step by step, though endometritis, metritis, peri- and parametritis, cellulitis terminating in peritonitis. Rupture of the vagina or uterus, extra-uterine pregnancy, accidents consequent to abortions, miscarriages, and the use of instruments, give rise to disturbances which pass into inflammatory conditions, and in many cases decide a peritonitis.

These are some of the etiological factors to be considered in discussing the origin of acute peritonitis. Other causes might have been mentioned, but the list already impresses one with the importance of judging insidious affections as causative of a more serious and many times a fatal disease.

Chronic peritonitis is often a sequel of an acute form; it may also follow upon an ascites, Bright's disease, tubercular or cancerous disease of some of the abdominal organs, conditions setting up long-continued irritations, etc.

The pathology of peritonitis is that of a serous membrane. But in no serous cavity can the propagation of bacteria take place so rapidly and so extensively as in the peritoneal. The ingress of micro-organisms is comparatively easy. The many avenues of approach, both internally and externally, the almost complete absence of protective structures, and the facility and ease with which neighboring processes extend into it, render it a fertile field for the reception and development of septic germs. Micro-organisms once within this cavity find in its congenial temperature—in the peristalsis of the intestine—in the circulation of the peritoneal fluids, and extensive development of the lymphatic system, agencies unequaled for the promulgation of their cause, and that cause is disease. It follows that most of the imflammations of the peritoneum are septic in character, due to the presence of micrococci.

Some of the more potent factors of septic inoculation are gunshot and incised wounds of the abdomen, perforating typhoid ulcers, puerperal fever, and gonorrheal infection. The onset of the inflammation is preëminently acute, and in a short space of time may become purulent,—or the absorption of sepsis may take place so rapidly as to produce septicemia before an inflammation can be developed. In the first case there follows a marked congestion and engorgement of the capillaries of some portion of the visceral or parietal layer of the peritoneum. This then becomes rapidly general, having its greatest intensity at the starting point of the inflammation. Ecchymosis and extravasations may occur, giving the peritoneum a mottled appearance. It becomes dry—lustreless—owing to the desquamation of endothelial cells, and so loses its natural glistening appearance. The first stage is now completed—the stage of invasion or inflammation.

Subsequent to the engorgement of blood-vessels denoting a vasomotor disturbance, there follows conditions of the vessel walls permitting the exudation of serum-lymph and leucocytes. Simultaneously we find swelling and multiplication of the endothelial cells.

The exuded lymph may form a coating on the free surfaces—producing a soft, thin yellowish layer—with this characteristic that it is highly adhesive. As a result, neighboring surfaces become adherent, the intestines are matted together or bound down to certain organs. We meet this circumstance very often in pelvic peritonitis. Serum may collect in the dependent portions of the abdomen, as in Douglas's pouch, and may become fibrinous, purulent, or hemorrhagic. In the majority of cases it becomes purulent. The visceral peritoneum is not dormant while the changes are going on elsewhere. The endothelial cells are separated and fall off in large patches, thus exposing the subperitoneal connective tissue which soon becomes infiltrated with serous fluid and leucocytes. The subserous, muscular, and mucous coats become edematous, swollen—paralysis of the muscular coat follows—giving rise to that pathognomonic symptom, meteorism.

Purulent peritonitis, if general, proves almost necessarily fatal.

We may have another form of acute general peritonitis, characterized by the absence of an exudate. This form has been denominated *cellular* peritonitis. Here the changes taking place in the endothelial cells are of prime importance; proliferation of these cells coating the surface of the peritoneum occurs, they become increased in size, and give the peritoneum a jagged appearance. The intestines are intensely hyperemic, but show no sign of a fibrinous exudate. Characteristic of this form of peritonitis is the absence of serum, fibrin, and pus.

In acute, local peritonitis we find the inflammation limited to a circumscribed area by the formation of adhesions, thus limiting further spread. Here we find the peritoneal cavity free from any exudate, and the intestines retain their smooth, glistening appearance.

In chronic peritonitis, both local and general, we may find the presence of adhesions matting the neighboring surfaces together, or it may show itself in the hypertrophy of the peritoneum, both visceral and parietal, or we may have the presence of a purulent serum, or blood in the abdominal cavity.

Tubercular peritonitis appears also under the chronic form, and may present three different varieties: 1, The predominance of adhesions; 2, of fibrin; 3, of serum, as ascites.

The acute form is, however, of paramount importance to the physician. Herein he is capable of showing his skill and tact as a clinician and therapeutist.

The next paper was that of Dr. H. R. Hopkins on the Symptomatology, Diagnosis, and Medical Treatment of Peritonitis.

[The MS. of this paper was not furnished for publication.—Ed.]

Dr. Herman Mynter read the concluding paper on the Surgical Treatment.

### SURGICAL TREATMENT OF PERITONITIS.

By HERMAN MYNTER, M. D.

The question assigned to me to-night is: The Surgical Treatment of Peritonitis. I might answer the question with one word, laparatomy, at least as a preliminary operation, and correctly, if by peritonitis is meant universal peritonitis, but wrongly if certain forms of local peritonitis, which may be opened by extra-peritoneal operations, are included. The question, too, implies that there is a medical treatment of peritonitis, and while acknowledging that occasionally cases of even diffuse peritonitis recover without operative treatment, I stoutly maintain that peritonitis *per se* is exclusively a surgical disease, can be treated successfully in the great majority of cases only by surgeons who are able to interfere operatively at a moment's notice. Too often surgeons are called in after the case by procrastination has become hopeless, and the unpleasant duty is thrust upon them of sharing the responsibility of signing the certificate of death. Some forms of peritonitis demand immediate operation, as those dependent upon gunshot wounds, perforations of the bowels, internal strangulations, etc. Other forms, particularly of local and circumscribed peritonitis or those septic forms following operations, are less urgent, and allow the institution of a medical treatment, at least for a short time. I am, therefore, unable to answer the question without stoutly referring to the etiology, and probably going over the ground again covered by the previous speakers. In regard to etiology I would distinguish between several classes.

1st. Those cases dependent upon rupture of a hollow viscus with entrance into the peritoneal cavity of the contents of the viscus, be it feces, urine, or bile, and be the rupture the result of ulceration, as in ulcers of cecum, duodenum and stomach, or of rupture of typhoid ulcers or else of accidents, as gunshot wounds, stabwounds, falls, etc.

These cases demand immediate operation,—at most waiting till the collapse disappears,—and all medical treatment and procrastination ought to be absolutely discouraged. We gain nothing by waiting; our object is to remedy the injury before the peritonitis has become universal. Opium, if given freely, may moderate the severe symptoms and lead us to the delusion, fatal to the patient, that he is improving; purging, according to Baldy's method (to be mentioned later), can only make things worse if the bowels be perforated, by making their contents thinner, so that the fecal material easier passes the perforation and enters the abdominal cavity. The depletion of the peritoneal blood-vessels, produced by the purging, can scarcely counterbalance the injurious effect of the increased peristaltic movements from the same cause, or the increased amount of fecal matter entering the abdominal cavity.

2d. As a second class I would mention those cases of diffuse peritonitis dependent upon the introduction of septic material into the abdominal cavity, be this the result of a laparatomy or herniotomy; the extension from, or perhaps the rupture of, a pyosalpinx, or a suppurating ovarian tumor; the rupture into the abdominal cavity of an abscess from somewhere else, as liver, kidney, or an extra-peritoneal abscess containing pus and nothing but pus; the extension to the peritoneum of septic inflammations from the womb during puerperisms or from gonorrhea; or, lastly, those rare forms, the cause of which we do not know, but which we call rheumatic peritonitis. Perhaps internal strangulations, ileus, volvolus, etc., etc., ought to be mentioned in this division. In these cases we may, perhaps, delay a little, and try different methods. The opium method, introduced and recommended by Austin Flint and Alonzo Clark, acts as a splint on the bowels, so to speak, by preventing peristaltic movements, and might help to produce a local peritonitis by giving adhesions a chance to form. But often we are lured to procrastination too long by the amelioration of the subjective symptoms. I am inclined to think that Baldy's purging method, published in the *Medical Standard* of October, 1889, is the more reliable method. It is recommended by Lawson Tait, too, particularly in cases of septic peritonitis. Besides operative treatment Baldy puts his main reliance on derivation to the alimentary canal by aid of strong cathartics, particularly those that produce copious, watery discharges, as the salts of magnesia. The treatment, to be sure, is in direct opposition to the teachings of most modern authors. In cases of universal peritonitis he gives ℥i. sulph. magnesia at a time, using four or five doses with proper intervals, till at least eight or ten or more copious, watery evacuations occur. If it is vomited up, he tries large doses of calomel. He declares that Flint and Clark had only failures by using cathartics, because they only tried to get one evacuation instead of many. Baldy insists upon that his results justify the treatment. The improvement, he says, follows immediately or not at all, and we do not run the risk of losing previous time as in the treatment with opium. The depletion of the blood-vessels following the copious evacuations more than counterbalance the possible injury from having the inflamed peritoneal surfaces rub against each other, the inflammation decreases, tympanitis and pain moderates, the exudation is easier absorbed, and adhesions are prevented by the peristaltic movements. Baldy does not recommend the treatment in all forms, nor maintain that it is always successful, but he believes that the surgeon by its use, or rather by its failure, will know when to operate, which he cannot do if opium is used. If no improvement follows, he advises immediate operation. He does not, of course, advise cathartics in those cases I have mentioned under class one, but refers more to the cases mentioned in class two, although he considers several of them demanding immediate operation, as suppurating ovarian tumors, pyosalpinx, abscesses of liver or other organs. He particularly recommends his method in peritonitis for which we can discover no cause, and states that in twenty-four or thirty-six hours we will then know what to do. If no improvement follows in that time, he advises to operate and to try to find and remove the cause. Irrigation and drainage must supplement every laparatomy on account of peritonitis.

Baldy's conclusions are:

1. If immediate operation is not indicated, use laxatives.
2. If unsuccessful, operate.
3. Remove the cause if possible.
4. And then use irrigation and drainage.

I have lately used this method in a case in which serious symptoms of peritonitis followed a prolonged and very difficult operation for an enormous strangulated umbilical hernia, containing besides omentum the whole transverse colon. The result was immediate and most gratifying, all the symptoms promptly disappearing. When he advises the same treatment in local peritonitis or perityphlitis, I cannot follow him, for reasons to be mentioned later.

3d. As a third class we might mention certain forms of chronic peritonitis, dependent often on tuberculosis or else on chronic inflammations with adhesions resulting from pelvic peritonitis, and forming incapsulated exudations. Uterus, ovaries and tubes may participate or not; in the latter case laparatomy alone is often successful without the removal of the sexual organs; in the former case the different operations for the removal of the uterus, ovaries and tubes may become necessary. Spaeth, (Annals of Surgery, August, 1889,) reports four cases of tuberculosis peritonitis treated at Prochownick's clinic in Hamburg by laparatomy. All were females, of ages ranging from thirty-two to forty-three years. The symptoms differed greatly, but tuberculous bacilli were found in all. The author considers this of importance, as many cases, which are considered of tuberculous origin, are simply peritonitis with a nodular formation,—a simple lymphoma of the peritoneum, and the prognosis therefore better. The first case died of collapse in five days, the second of acute phthisis in three months, and the third and fourth of intestinal tuberculosis.

The author's conclusions are:

1. In primary tuberculosis of peritoneum, without implication of other organs, laparatomy may act as a curative agent, and is to be recommended.

2. If tuberculosis be present in the female sexual organs too, operative treatment has not given any satisfactory results.

3. If the tuberculosis is due to a tuberculous enteritis, the operative treatment is only palliative.

4. In genital tuberculosis unaccompanied by peritoneal tuberculosis, early radical operation is to be urged.

Prochownick, himself, thinks that much of the success claimed for operative interference in tuberculous peritonitis, is due to errors in diagnosis. His conclusions are, that in cases of chronic peritonitis in which extensive omental adhesions exist, laparatomy is indicated. He also calls attention to the fact that chronic peritonitis, in many cases, produces the symptoms for which castration is practised. Schroeder and Martin regard the accompanying chronic peritonitis as the causative factor in the majority of cases of öophoritis and diseases of the tubes, and, therefore, advocate conservative treatment. Laparatomy is often followed by adhesions, and these may be prevented by loosening any adhesions already present, by avoiding strong antiseptics, by removal of contused omentum, strict toilet of the peritoneum, and lastly, by not giving opium before or after operation.

It is difficult for me to see what influence a simple laparatomy, even when irrigation and drainage are added, could possibly have in tuberculous peritonitis, pure and simple, and I cannot help believing with Prochownick, that in successful cases there must have been errors of the diagnosis. The peritoneum is very similar to the synovial membrane of the knee-joint, for instance. Does anyone believe that incision, followed by drainage and irrigation, would be of any use whatever in a case of tuberculous synovitis. Would not all scorn such an idea and advocate the radical treatment of arthrectomy? That incision, irrigation, and drainage, may be an excellent and curative treatment in chronic forms of synovitis without tuberculosis, will not be doubted by any surgeon. I never saw a case of tuberculous peritonitis myself, or failed to recognize it if I did, but from theoretical reasons I am strongly inclined to believe that successful cases of laparatomy in primary tuberculosis of the peritoneum depend upon errors of diagnosis. That laparatomy may be successful if tuberculous sexual organs, primarily affected, be removed, stands to reason, and it is an entirely justifiable operation, provided all diseased tissues be removed. Spaeth's statement that operative treatment fails or is only palliative, if besides tuberculous peritonitis tuberculosis of the female sexual organs or tuberculous enteritis be present, rather proves that my conclusion is correct. That operation is useless, unless the diseased tissues be removed in toto. It would, in my estimation, do a patient as much good to remove the tuberculous sexual organs and leave a tuberculous peritoneum behind, as it would do good to remove a local tuberculosis focus from the lower epiphysis of the femur and leave the tuberculous synovial membrane behind.

4th. As a fourth class, I wish to mention certain forms of local peritonitis, referring particularly to perityphlitis, because several questions of importance to the surgeon are still being discussed, and because I consider the treatment by purging, advocated by Baldy, directly injurious in cases of circumscribed perityphlitis, and little less than fatal if universal peritonitis be present, from either primary perforation of the cecum or secondary perforation of a local abscess. As I expect to read a paper shortly in the Buffalo Medical Association on this subject, I will here only mention, that in all cases of perityphlitis

I consider the opium treatment of the greatest importance. While Dr. Rochester, for instance, lost seventeen out of twenty-three cases, I have had eleven recoveries in eleven cases, five after operation. The important point is to keep the patient at rest, bodily and mentally, in order to prevent, if possible, perforation into the abdominal cavity, and in order to keep the abscess circumscribed until you can open it by extra-peritoneal operation, after Gurdon Buck's method.

If perforation occurs, primarily or secondarily, laparatomy is the only remedy, and ought to be done promptly, at most waiting till the symptoms of collapse following the perforation have disappeared. All surgeons agree now upon this point, the only difference being in regard to the time when it ought to be performed. Enough cases of early successful operation are on record to convince the most sceptical of its advantage, and still more convincing are the unfortunately still frequent cases of death following the expectant or medical treatment of perforative perityphlitis. You all remember the unfortunate death, under medical treatment, of a young and promising physician here in Buffalo, a couple of years ago. I shall mention but one point more: considering the high percentage of relapses in perityphlitis, about twenty-three per cent., and considering that a previous mild attack is no guarantee that a future attack will be equally mild, the question has necessarily been raised, whether it is not preferable in cases of relapsing perityphlitis to operate while the patient is convalescent, instead of letting him run the risk of a further and perhaps fatal attack. "A positive cure," Krafft says, "free from relapses, is only possible with operative treatment." No case is on record in which relapse has occurred after once a circumscribed abscess has been operated.

Krafft believes that in a few years every perityphlitic abscess will be operated upon, even after the disappearance of all symptoms, and this appendix ligated and cut off. Bull considers it still doubtful whether we ought to make laparatomy in relapsing cases. It is our English confrères, especially Lawson Tait, who have led the way. In the *British Medical Journal*, of October 5, 1889, Lawson Tait reports a case of relapsing perityphlitis successfully treated by operation. The patient had had repeated attacks of perityphlitis for half a year, three in all, the last one being the most severe. During each attack, the characteristic egg-shaped tumor had been felt, and was still distinctly marked. An incision, three inches long, was made above the cecum and a suppurating cavity opened on the outside of the cecum. The appendix was discovered swollen to three times its size. It was split open, some purulent fluid evacuated, and a concrement, felt in the upper part, pushed into the cecum with a catheter.

Although the operation had been made extra-peritoneally, the abdominal cavity was opened during the manipulations, but the opening was closed with sutures. The appendix was not removed, but a drainage-tube introduced into it, and left in position for a couple of days. The patient recovered without bad symptoms. In similar cases, Lawson Tait has twice removed the appendix, finding it thickened, swollen and suppurating, but he thinks the risk is increased by this proceeding and the removal unnecessary, preferring to open and drain it.

Treves mentions one case, who had had fourteen attacks and been in bed twelve months; he, too, recovered by operation. Treves favors operation between the attacks, too. I have so far seen no case in which I would recommend this proceeding. Although the percentage of relapses is high, twenty-three per cent., it is in my opinion scarcely high enough to justify such a serious operation, except in unusual cases with continued relapses, which make the life of the patient such a burden and misery to himself, that he prefers to take any risk in order to get well permanently.

Dr. M. D. Mann, in opening the discussion, disagreed with the second essayist's statement that pain, tympanitis, vomiting, etc., are always present in peritonitis. He mentioned lymphatic or septic peritonitis as having none of these symptoms except the small, rapid pulse, and diarrhea. These are most insidious and fatal cases, and the post mortem reveals the abdomen full of purulent fluid. With the third essayist's remarks on tubercular peritonitis Dr. Mann did not agree. He quoted a reported case of Dr. Van DeWarker; the patient's abdomen having been opened and drained for tubercular peritonitis, and two years later the patient was absolutely robust. Dr. Mann had two of his own experiences to report. Some time ago he washed out the abdominal cavity, having found the parietal peritoneal layers dotted with tubercles, and the intestines matted down by adhesions. So far this patient has seemed to enjoy immunity from the destructive ravages of tuberculosis. In the second case he operated for abdominal dropsy and found the peritoneum studded with tubercles. He removed one tube and ovary. The patient is not failing, although the dropsy returned. In both these cases he did not microscopically examine the tubercles.

In cases of general peritonitis, Dr. Mann believes with our best efforts the chances are slim for the patient. He mentioned Greig Smith's method of operating for such cases; namely, to open the abdomen and to irrigate the following day in order to avoid extreme shock. While he does not believe in opium treatment, he has found salts not always reliable for cathartic effect and sometimes nauseating.

Dr. Chas. G. Stockton believes there is a distinct field in peritonitis for both surgeon and physician. In traumatic cases the surgeon has his field, while in cases of spontaneous origin the physician, he believes, finds his best results from following the teachings of Dr. Alonzo Clark, as set forth by Dr. Hopkins. Dr. Stockton refrained from further discussion of the question of treatment because Dr. Hopkins had gone away after reading his paper. He wished to state, however, that he objected to the radical steps of surgery as advocated by Dr. Mynter.

Dr. John Parmenter sincerely believes the opium treatment will be discarded soon, and he instanced the treatment of pleurisy as more rational without opium. As for the treatment of laxatives he believes that many cases of alleged cure therefrom are really cases in which no peritonitis existed. When pus was present in general peritonitis he hoped it would not be ascribed to youthful boldness if he stated his belief in opening the abdomen, on the same principles that a suppurating joint is opened.

Dr. John Pryor said that evidently the physician must choose safe, middle ground, between the extreme views which the discussion had brought out. He believed that one should distinguish between the forms and conditions with regard to etiology, and the necessity for medicinal treatment, or operative interferences. It had been clearly demonstrated that we have two classes of cases. One where the cases were distinctly surgical, and another, constituting a general peritonitis, with no apparent cause, where to attempt laparatomy would be folly. Dr. Hopkins's positive assertion that general peritonitis was sometimes spontaneous in origin, he believed dangerous, because the idea is misleading and the opinion in opposition to the advanced knowledge of the times. The number of cases of so-called idiopathic peritonitis, grows steadily smaller with the increasing knowledge of the etiology and pathology of the disease. The arguments offered to support the theory of spontaneous disease, is at least disappointing. A conviction is announced, and an authority quoted whose clinical and pathological researches were made before recent investigation, added so much to our knowledge of the causative factors and surgical aspect of the subject.

Peritonitis arising without a definite known cause, manifestly calls for medical treatment, but the cause should be searched for in the hope of removing it, and any theory which leads away from this plan is unscientific, and may be harmful. The time for operating is neither too early nor too late. The treatment by opium, according to the method of Alonzo Clark, which has been so strongly recommended here this evening, was generally employed some years ago, but he had thought that it was passing away ; apparently it still existed as a relic of the days of empiricism. Recent discussions and a consideration of modern methods, make it questionable whether it is rational to administer opium until the respirations remain at eight or ten to the minute for several days. Narcosis, of a marked degree, is added to the depressing effects of diseased actions. Pain can be quieted by morphine in the same manner as in other affections. In no other disease or condition is opium or its preparation employed as a specific remedy, and according to arbitrary rules. By securing deep and prolonged narcosis, the symptons are obscured or hidden, particularly those of a subjective nature, thus preventing or delaying a clearer appreciation of the local condition, which otherwise might demand radical or prompt action for its relief. He regretted that Dr. Hopkins had left the room, as he desired to discuss some of his statements relating to symptomatology. Some of them were novel and peculiar, and he did not think they should appear to have won acceptance by silence. The diagnostic value of the pulse in diseases of the serous membranes had been over-estimated, and as a generalization he did not believe it was clinically correct. The essential differences noted in the character and forms of peritonitis were enough to reveal a fallacy without citing the dissimilarity in pleurisy, pericarditis, or referring to intrinsic influences and stages of disease. The pulse described is found in low forms, particularly of a septic nature, like those mentioned by Dr. Mann, and is noticed in other conditions when there is sufficient systematic derangement to produce it. He would like to express disagreement with other views which had been advanced but refrained at this time.

Dr. Jas. W. Putnam related the history of a case of Dr. Burwell's in which quick relief of all the symptoms followed bleeding. In the strong and plethoric he thought venesection an important procedure in treatment.

In closing the discussion Dr. Mynter repeated his statement that opium masks the disease and deludes the physician. He also announced his belief that Dr. Mann had erred in diagnosticating tubercle in his two cases, the more so since he had made no microscopical examination to verify. Dr. Mynter believed recovery impossible if tubercular nodules were left.

Under presentation of specimens, Dr. Krauss exhibited a specimen of marked atheroma of the aorta and an hypertrophied heart with thickened, insufficient valves.

Dr. Mann gave the following history of a case: Several months after laparatomy a patient of his developed a large ventral hernia. Going without an abdominal support, and suddenly stooping, the abdominal wall burst open in the line of the cicatrix, exposing the intestines. An antiseptic dressing was applied until next day, when the line of incision was more fully opened and resewed, stitches being also used to unite the muscle fascia. The patient made a good recovery. Dr. Mann emphasized the importance of suture of the fascia to prevent hernia. In the original operation in the above case he failed to follow his usual procedure.

